# 2024 transplant AI symposium: key AI models shaping the future of transplant care

**DOI:** 10.3389/frtra.2025.1723799

**Published:** 2026-01-05

**Authors:** Annabel Koivu, Ghazal Azarfar, Saba Maleki, Aman Sidhu, Mamatha Bhat

**Affiliations:** 1 Department of Medicine, University of Toronto, Toronto, ON, Canada; 2 Transplant AI Initiative, Ajmera Transplant Centre, University Health Network, University of Toronto, Toronto, ON, Canada; 3 Toronto General Hospital Research Institute, University Health Network, Toronto, ON, Canada; 4 Division of Gastroenterology & Hepatology, Department of Medicine, University of Toronto, Toronto, ON, Canada; 5 Vector Institute, Toronto, ON, Canada

**Keywords:** transplant, artificial intelligence (AI), solid organ transplant, liver transplant, lung transplant, cardiac transplant

## Abstract

Experts in transplantation medicine and AI innovation came together to showcase advancements in AI applications with the potential to improve transplant outcomes. Ethical deployment, consolidation of multimodal data and supporting clinical decision making were among the themes addressed. Experts presented foundational models such as MedSAM for universal medical image segmentation, scPGT for single-cell genomics and Clinical Camel for clinical decision support, each demonstrating high capability and adaptability across transplant specialities. Experts highlighted future directions, considerations, and challenges for integrating these tools into clinical practice in an ethical and safe manner. We will summarize these themes as discussed at the Ajmera Transplant Centre's second annual Transplant AI Symposium.

## Graphical Abstract

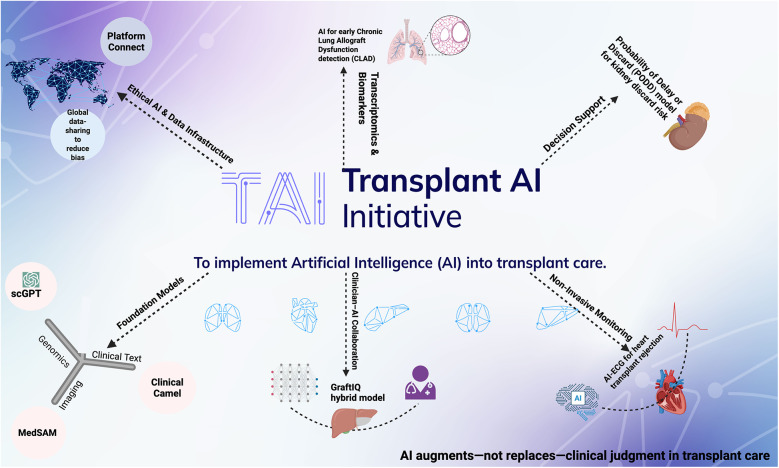

## 1 Introduction

The Ajmera Transplant Centre hosted its second annual Transplant Artificial Intelligence Symposium in Toronto in 2025, building on the success of the 2024 symposium, which showcased emerging applications of AI in transplant medicine while underscoring the challenges and considerations critical to their safe and effective implementation. Artificial intelligence (AI) is rapidly transforming medicine, with solid organ transplantation emerging as a key area of interest. Transplantation involves complex decisions based on vast, varied data—spanning patient selection, organ allocation, perioperative care, and long-term management. Traditional methods often fall short in handling this complexity. AI and machine learning can integrate high-dimensional data, uncover patterns, and generate predictive insights to improve outcomes—from identifying candidates and matching organs to monitoring grafts and tailoring immunosuppression. However, challenges remain, like limited data, transparency issues, and risks of exacerbating disparities. Responsible development and patient-centred approaches are essential.

## 2 Symposium highlights

### 2.1 Dr. Halamka's vision

Dr. John Halamka, president of the Mayo Clinic Platform, highlighted the potential for AI to revolutionize transplant medicine when developed responsibly and equitably. He stressed the importance of ethical deployment of AI, noting that algorithms must be designed with transparency from the start. He stated that the healthcare industry needs to transition to focus on the “algorithmically underserved” (1), individuals who do not generate enough data or are underrepresented in existing datasets, to ensure that innovations benefit all (2). There is a need for strong guidelines and validation to purge bias in AI models. He described the need for rigorous multi-site testing and prospective studies before clinical use, to be sure there is evidence of its accuracy and equity (3). He urged that AI should be as trustworthy and compassionate as the clinicians using it, augmenting physicians' clinical judgment rather than replacing it (4). Halamka's guiding principle is that AI should be used to enhance fairness in medicine, not just speed up processes.

Dr. Halamka's keynote speech described a hopeful roadmap for the future of AI in transplant medicine. It is well known that for AI, the more data the better. Therefore, a multimodal data infrastructure, integrating many types of health data (clinical notes, laboratory values, diagnostic imaging, and genomics, etc) on a worldwide scale would be paramount. Halamka introduced a global data-sharing platform called “Platform_Connect” to fuel AI innovation (5). Platform_Connect is a federated network that links health systems across the world, allowing each partner to contribute clean and de-identified data while retaining control of their data behind secure firewalls (5). Each participating health system member brings an archive of high-quality data on complex and rare conditions, which is vital for developing robust AI models. By consolidating data from vastly diverse groups of patients, the platform helps reduce bias and improve the generalizability of AI findings. Some health networks contributing data to this partnership include University Health Network (UHN) in Toronto, SingHealth in Singapore, Sheba Medical Center in Israel, and Hospital Albert Einstein in South America. This allows researchers to develop AI tools that learn from vast and diverse data, revealing more widely representative patterns that could not be discovered by individual institutions.

Dr. Halamka then described how these recent advancements in AI specifically impact transplant medicine. Transplant care involves a complex interplay of donor, recipient, genetic and imaging data, which AI can rapidly integrate and analyse data to predict organ compatibility, rejection risks, and personalize post-transplant care (6, 7). For example, AI could analyse historical transplant cases from the Platform_Connect network to identify early warning signs of rejection or factors that lead to better long-term graft survival. As AI is only as good as the data it learns from (8), a model trained on records across different regions helps models learn what is universal rather than specific to one group. This diversity is critical in transplant medicine, where genetic and cultural factors can influence outcomes. Ultimately, AI may enhance organ utilization and long-term patient survival while improving overall quality of life.

Dr. Halamka then highlighted the cautions and challenges that must be addressed as these AI tools become integrated into medical practice. Safeguarding data privacy remains a paramount consideration, especially given the sensitive nature of health information and the added complexities of sharing data across institutions and international borders. He described Mayo Clinic's approach of “Data Behind Glass”, a security architecture that keeps patient data encrypted and on local servers while allowing algorithms to run across sites (9). Additionally, de-identifying data is complex, resource-intensive, and impossible to achieve perfectly. There remains a balance between removing identifiers and retaining enough detail for research. Technological readiness does not necessarily match clinical adoption: “innovation depends upon a perfect storm of technology, policy and culture” (2). Institutions differ in their comfort with AI tools, and some clinicians are cautious about trusting opaque “black box” algorithms (10, 11). This can be combated with transparency, peer-reviewed validation, and involving clinicians in AI design and training. To enable the safe and effective integration of AI into transplant medicine, there must be interdisciplinary collaboration among technology developers, clinicians, policymakers, and patient stakeholders, and issues of data privacy and algorithmic bias must be addressed.

### 2.2 Foundation models for optimization of transplant medicine

Dr. Bo Wang, Chief AI Scientist at UHN, discussed generative AI and foundation models in medicine, emphasizing their ability to generalize across complex biomedical domains including genomics, imaging, and clinical text. He discussed how these large-scale, pre-trained models, including scGPT, MedSAM, and Clinical Camel, can be fine-tuned for specific healthcare tasks, offering improved data efficiency and insight generation. Dr. Wang also underscored the importance of clinical validation and thoughtful integration, given ongoing concerns about model bias and explainability.

scGPT, a foundation model by Dr. Wang's team draws an analogy between language and cellular biology (in which texts comprise words; similarly, cells are defined by genes) (12). The model was pre-trained on a library of over 33 million single cells, enabling it to “distil critical biological insights” about genes and cells (12). Dr. Wang enlightened the group about scGPT and that it can then be fine-tuned (via transfer learning) for diverse downstream tasks without needing enormous new datasets. For instance, scGPT can be adapted to perform cell type annotation, integrate data across batches and omics modalities, predict perturbation responses, and infer gene networks, all with impressive accuracy (12). This underscores the data-efficiency of foundation models: after broad pre-training, relatively little additional data is required to achieve strong performance on specific biomedical problems (12). Clinically, such a model could accelerate discoveries in transplant immunology—for example, helping researchers characterize immune cell subtypes in transplant biopsy samples, potentially accelerating the development of improved therapies to avoid and or manage allograft rejection (13, 14). The foundation model approach thus offers a powerful tool to extract insights from complex single-cell data, which has the potential to be pivotal for improving transplant care.

Transitioning to medical imaging, Dr. Wang presented MedSAM, a foundation model for universal medical image segmentation that outperforms traditional algorithms by working across multiple imaging modalities and disease types. Trained on over 1.5 million image–mask pairs spanning 10 different modalities, MedSAM demonstrates strong generalizability and accuracy in segmenting organs, tumours, and other structures in diverse image types such as CT, MRI, and pathology slides (15). Dr. Wang underscored its clinical value in enhancing diagnostic efficiency and consistency through automated, standardized segmentation. In the setting of transplant medicine, such a tool might provide assistance in identifying rejection on imaging studies or segmenting donor organs for surgical planning. The efficiency and consistency of a foundation model like MedSAM can reduce radiologic workload and potentially uncover subtle imaging biomarkers of graft health, contributing to more personalized transplant patient medicine management (16). Furthermore, Dr. Bo Wang addressed an open source large language model his team developed that is specifically tailored for medical text and decision support tasks called Clinical Camel (17). Clinical Camel was devised by altering and fine-tuning Meta's LLaMa 2 using a QLoRA method allowing it to achieve expert level performance on clinical specialty board exams and medical licensing examinations (17). The key innovation is the use of medical dialogue which improves expertise and accuracy for clinical utility but the need for ongoing human evaluation is necessary to ensure the model's safe, fair and effective.

Dr. Wang's emphasized that transplant medicine is uniquely positioned to benefit from generative AI and foundation models given the field's inherently complex, multimodal data and need for individualized management of transplant patients. Foundation models such as MedSAM, scGPT and Clinical Camel can augment diagnostics, enable personalized prognostication and streamline workflows of clinicians by integrating diverse and complicated data. He further underscored the need for vigorous validation, transparency and clinician involvement to ensure these tools are safe, ethical and meaningfully improving outcomes for transplant patients. Key models and platforms discussed across genomics, imaging and clinical decision support are summarized in Table 1.

**Table 1:** Comprehensive overview of the key artificial intelligence models and platforms presented by leading experts at the 2025 transplant AI symposium.

Speaker	Model Name	Domain	Specific Functionality	Application in Transplant Medicine	Performance Metrics	Validation/Deployment
Dr. Halamka	Platform_Connect	Infrastructure	Federated data-sharing platform for AI development	Enables global multimodal data integration for transplant AI	Not model-specific	Deployed across Mayo Clinic, UHN, SingHealth, Sheba, etc.
Dr. Wang	scGPT	Genomics	Transformer-based single-cell transcriptomics analysis	Immune cell profiling and biomarker discovery for graft rejection	High-resolution clustering; validated on transplant datasets	Pretrained on 33M cells; fine-tuned for transplant immunology
Dr. Wang	MedSAM	Imaging	Vision transformer for medical image segmentation	Histopathology slide analysis for graft assessment	Dice coefficient > 0.85 on biopsy images	Trained on 1.5M image-mask pairs; multi-modal validation
Dr. Wang	Clinical Camel	Text	LLM for clinical decision support using EHR data	Prediction of graft failure and patient outcomes	AUROC 0.92; expert-level exam performance	Fine-tuned from LLaMa 2 using QLoRA; open-source deployment
Dr. Halloran	Transcriptomic CLAD Model	Genomics	ML-based transcriptomic analysis of lung biopsies	Early detection of chronic lung allograft dysfunction (CLAD)	AUROC ∼0.87 for CLAD prediction	Multicenter biopsy study; validated against histology
Dr. Segev	PODD Model	Decision Support	Random forest model for kidney discard prediction	Identifies high-risk kidneys to reduce discard rates	C-statistic ∼0.87	Validated on national registry data; online calculator available
Dr. Sharma	GraftIQ	Hybrid ML	Deep learning + clinician-informed Bayesian fusion	Classification of liver graft injury types	AUC improved from 0.885 to 0.902	Developed with hepatologist input; interpretable predictions
Dr. Adedinsewo	AI-ECG	Cardiology	CNN model for ECG-based rejection detection	Non-invasive monitoring of heart transplant rejection	AUC ∼0.84; sensitivity 95%	Retrospective study of ∼7,500 ECG-biopsy pairs; EHR integration

### 2.3 Potential of transcriptomics and biomarkers in transplant medicine

In his Transplant AI Symposium keynote, Dr. Kieran Halloran emphasized the power of transcriptomics, high-throughput gene expression analysis, to uncover hidden molecular signals of injury and rejection in transplant patients (18). Traditional monitoring of transplanted organs often relies on physiologic metrics and biopsy histology, which in lung transplantation are notoriously limited in predicting chronic rejection (18). Dr. Halloran highlighted that chronic lung allograft dysfunction (CLAD), the fibrotic “chronic rejection” that affects about half of lung recipients within five years, typically develops without clear early warning signs on routine tests (18). Using AI to analyse biopsy transcriptomes, however, can reveal the underlying biology of CLAD and enable earlier detection. For example, Halloran's team showed that gene expression profiles in lung biopsies from patients who eventually develop CLAD have a distinct “wound-healing” signature—characterized by upregulation of injury-response and fibrotic pathways (e.g., HIF1A, SERPINE2, IGF1) rather than just inflammation (18). This indicates that CLAD is driven by a tissue remodelling and dedifferentiation at the molecular level, which routine histology often cannot detect (18). By using machine-learning trained on hundreds of biopsies, they have been able to develop a transcriptomic test that predicts future CLAD with high accuracy (area under the curve ∼0.87 after adjusting for time post-transplant) (18). Also, notably the genes that flag early CLAD risk also correlated with the development of graft failure and disease severity underscoring the potential of AI transcriptomic diagnostics that can expose early pathological changes and provide an opportunity of proactive intervention.

Dr. Halloran's keynote also stressed a shift toward biomarker-driven stratification of transplant patients, the potential for using AI to stratify patients into high-risk vs. low-risk for CLAD based on biomarkers and transcriptomics. He highlights that similar to other clinical issues in transplant medicine with complex diagnostic criteria, there is no single test sufficient for the diagnosis of CLAD. Therefore, using AI helps aid in clinical decision making by combining clinical data with molecular and cellular biomarkers to improve predictive power. For example, AI tools that incorporate diverse and complex multimodal data such as donor-specific antibody levels, donor-derived cell-free DNA, and gene expression indices into a unified risk score for graft rejection or failure have great potential (19). For example, Dr. Phillip Halloran, clinician scientist and nephrologist, has demonstrated that a molecular microscope approach (whole-genome microarray analysis of biopsy tissue with machine learning) can identify patients with subclinical rejection risk (20). Similarly, Dr. Kirean Halloran's multicentre study found that lung transplant recipients whose biopsies exhibited a T cell–mediated rejection (TCMR) gene-expression “archetype” faced higher graft-loss risk despite normal histology, suggesting transcriptomic AI models could detect otherwise-invisible rejection processes and flag high-risk patients early (21). By stratifying patients in this way, AI can help personalize care: clinicians might alter monitoring strategies or immunotherapy regimen in a patient with worrisome molecular signals, while avoiding unnecessary interventions in lower-risk patients. Dr. Kirean Halloran emphasized the potential of AI to analyse extensive biomarker data which were previously underused.

### 2.4 Supporting clinical decision making

Dr. Segev, director of the Center for Surgical & Transplant Applied Research at NYU Langone Health, emphasized that the future of transplant medicine depends on deploying big data and AI where they offer tangible benefits—not simply because they are emerging technologies. Dr. Segev spoke about how AI can capture unique insights by analysing large and complex registries and databases, learn from patterns within the data, better than humans could alone, and use this acquired knowledge to predict outcomes such as graft failure, rejection, or patient survival. For example, his team developed a risk prediction tool using a random forest algorithm to estimate an individual patient's post-transplant survival benefit based on the combination of donor organ quality and candidate health status (22). The analysis revealed that even patients with higher medical risk (e.g., older or comorbid recipients) still gained substantial survival benefit from transplantation, even when receiving so-called “marginal” organs (22). Similarly, by analysing national data, Dr. Segev's group created the Probability of Delay or Discard (PODD) model to identify kidneys at high risk of discard (23). This model proved highly accurate (C-statistic ∼0.87) in predicting which organs might otherwise go unused (23). Organs labelled as high-risk by PODD (PODD > 0.6) were disproportionately ignored by many centres—more than 60% of centres never transplanted these kidneys (23). Analysis of offer patterns showed 35% of high-PODD offers went to centres that never accepted them, indicating significant clustering in utilization (23). This suggests that proactively directing high-PODD kidneys to centres with demonstrated willingness to use them could substantially reduce organ discard rates, increasing transplant opportunities without lowering quality standards (23). Dr. Segev highlighted that AI could serve as a valuable assistant in complex transplant decisions making organ allocation more objective and efficient while reducing discard of valuable organs. He noted that online tools now enable real-time, individualized organ offer assessments, move beyond a one-size-fits-all approaches (22).

This shows how AI-driven insights have the potential to inform policy, serve as decision-support algorithms, mitigate human biases, and expand organ use. The goal is not to replace expert judgment, but to augment it with evidence-based recommendations, “the AI agent in the room” guiding deliberations with objective data. This is crucial as there are persistent disparities in transplant access and outcomes across different patient demographics and regions, and strategic use of AI can help identify and address these gaps and inequities. Dr. Segev's outlined a future of AI in transplant medicine where human clinical expertise and artificial intelligence work hand in hand to advance transplantation into a new era of data-informed, optimized care.

### 2.5 Integrating clinical expertise into multimodal AI tools

Dr. Divya Sharma, a machine learning expert at York University, described how integrating clinician expertise with AI enhance diagnostic accuracy. Her team developed an interpretable deep learning model trained on clinical and laboratory data to classify liver transplant outcomes into six graft injury categories (acute cellular rejection, autoimmune hepatitis, biliary obstruction, congestion, HCV or MASH) (24). The model generated individualized risk probabilities and highlighted key clinical drivers for each prediction, avoiding a “black-box” approach. The deep learning model achieved an AUC >0.75 for all categories, again showing the potential of AI for improving prognostication in transplant medicine (24). However, Dr. Sharma noted how machine learning models face limitations in transplant decision-making, particularly in capturing nuanced clinical context. She discussed cases where the stand-alone ML model misclassified liver graft injury (e.g., acute rejection vs. metabolic injury) that hepatologists easily distinguished. AI and ML tools can miss clinical nuance that physicians acquire from years of expertise and exposure to the specific population. To address this, Dr. Sharma introduced a clinician-informed, multimodal model (“GraftIQ”) that integrates clinician-derived decision rules with the neural network outputs via Bayesian probabilistic fusion, improving overall AUC from 0.885 to 0.902 (24). There is power in supplementing AI-derived prognostic predictions with clinical expertise for enhanced predictive performance and accuracy, showing that AI-clinician hybrid models should augment rather than replace physician judgment.

### 2.6 AI supported non-invasive monitoring

Dr. Demilade Adedinsewo, a Mayo Clinic cardiologist discussed how deep learning can detect early signs of heart transplant rejection non-invasively. Currently, surveillance for acute cellular rejection in patients with heart transplants requires serial endomyocardial biopsies (EMB), especially in the first year. However, EMB is invasive, extensive and associated with considerable risk. This has resulted in an interest in the development of less invasive methods. Existing blood-based surveillance tools—such as gene expression profiling (AlloMap) and donor-derived cell-free DNA—have modest accuracy (area under curve ∼0.68–0.77 for rejection) and have not yet been broadly adopted (25). Due to these simulations, Dr. Adedinsewo's team has worked to leverage the power of AI to improve monitoring of heart transplant patients. An example is an AI-enhanced electrocardiogram (AI-ECG) tool to detect acute cardiac allograft rejection non-invasively. 12-lead ECGs are already obtained in the follow-up of transplant patients, and severe rejection episodes often show subtle ECG patterns indiscernible to clinicians but might signal rejection; deep learning can exploit these features. Building on prior AI-ECG successes in cardiology, such as models detecting asymptomatic left ventricular dysfunction (26) and silent atrial fibrillation in sinus rhythm (27), Adedinsewo's team trained a convolutional neural network to recognize moderate-to-severe acute cellular rejection from ECG data (25). In a multi-centre retrospective study of ∼7,500 ECG-biopsy pairs, their model achieved an impressive AUC ∼0.84 with 95% sensitivity for rejection in an independent test cohort (25). This AI-driven approach could transform transplant care by enabling rapid, non-invasive, and even remote monitoring for rejection. At Mayo Clinic, such tools are already integrated into electronic health records, providing real-time predictions and supporting timely clinical decisions.

## 3 Discussion

Across disciplines—from cardiology and hepatology to pathology and genomics—AI tools are already reshaping clinical workflows, enhancing diagnostic precision, and enabling more personalized, data-driven care. While the future of AI in transplant medicine is optimistic, implementation through responsible and cautious measures is paramount. Professionals in the field continue to stress the potential for AI to dehumanize patient care (28). In transplant medicine, where longitudinal relationships and empathy are essential to patient trust and directly related to quality of care, human connection is essential. A sustainable vision for transplant AI requires a hybrid model of shared responsibilities where algorithms handle routine, data-intensive tasks and physicians can concentrate on comprehensive patient-facing care. Similarly, the cybersecurity risk and data privacy concerns, particularly as federated networks expand across institutions, is significant (29). In transplant medicine, where patient data is often highly sensitive information, data privacy issues could have critical implications for patient confidentially and institutional trust and credibility. Strong encryption, regulatory frameworks and monitoring will be essential to implementing AI into clinical medicine while safeguarding privacy and trust.

The future of AI in transplant medicine focuses on building trustworthy, equitable systems that align with real-world clinical needs. Yet, while the technological progress is impressive, professionals in the field consistently stress the need for careful validation, clinician involvement, and ethical implementation to ensure these tools translate into meaningful improvements in patient outcomes. As the field evolves, interdisciplinary collaboration and robust evaluation will be essential to realizing the full promise of AI in transplantation.

## Data Availability

The original contributions presented in the study are included in the article/Supplementary Material, further inquiries can be directed to the corresponding author.
